# Three-dimensional volumetric analysis of bone regeneration following jaw cyst enucleation with and without an autologous albumin gel-platelet-rich fibrin mixture (Alb-PRF): a randomized controlled clinical trial

**DOI:** 10.1186/s12903-025-06027-w

**Published:** 2025-05-08

**Authors:** Mohamed Magdy Elsayed Mohamed Shokry, Lydia Nabil Fouad Melek, Tasneem Ahmed Amer

**Affiliations:** 1 Faculty of Dentistry, Alexandria University, Champlion Street, El-Azarita, Alexandria, Egypt; 2 Department of Oral and Maxillofacial Surgery, Faculty of Dentistry, Alexandria University, Champlion Street, El-Azarita, Alexandria, Egypt

**Keywords:** Three-Dimensional Volumetric Analysis, Bone Regeneration, Centrifugation, Albumin Gel-Platelet-Rich Fibrin Mixture, Spontaneous Healing, Bone Density, Cone Beam Computed Tomography, Jaw Cysts

## Abstract

**Introduction:**

The presence of an osseous cavity after cyst enucleation is a clinical challenge that needs to be considered. Using pure autologous concentrations of platelets, platelet-rich fibrin (PRF), as a graft material after cyst removal has shown promising effects. However, PRF has limitations in terms of durability, as it usually resorbs within 10–14 days, thus Mourão et al. introduced a new technique for PRF preparation to obtain an albumin gel-platelet-rich fibrin mixture (Alb-PRF), a new autologous material, that can remain stable for 4–6 months with the ability to regenerate bone. This research aimed to evaluate the effect of Alb-PRF on bone regeneration after jaw cyst enucleation via 3-dimensional (3D) volumetric analysis.

**Methods:**

Twenty participants, with jaw cysts, were split into two groups. The Alb-PRF group included 10 individuals treated by enucleation and Alb-PRF application, and the control group included 10 individuals treated conventionally by enucleation without any additives. Cone beam computed tomography (CBCT) was conducted immediately following surgery (T1) and six months later (T2) to measure the volume of the residual bone cavity and the mean bone density of the regenerated bone using On-demand 3D viewer. Paired t test was used to compare the postoperative immediate results with the post-6-months results, whereas Student t test was used to compare the Alb-PRF group with the control group.

**Results:**

At the 6-month follow-up, the volume of the residual bone cavity had declined and the bone density had increased significantly in both the Alb-PRF group and the control group (P_1_ < 0.001) compared with the immediate postoperative values. Although the changes in volume and density were greater in the Alb-PRF group than in the control group, there was no a noticeable difference between the two groups. (*P* = 0.821) and (*P* = 0.533), respectively.

**Conclusion:**

There was no difference in bone regeneration between Alb-PRF and conventional blood clots after jaw cyst enucleation.

**Trial registration:**

The trial was retrospectively registered at the Clinicaltrial.gov registry (Registration ID #NCT05658900). It was first submitted on 12/12/2022 and first posted on 21/12/2022.

## Introduction

A cyst is a diseased cavity containing liquid or gas. It has an internal lining of epithelial tissues derived from tissues producing the tooth or tissues other than the tooth-producing tissues. Cysts may cause long-term swelling of the jaw [1].

Cysts enlarge gradually until they cause bone thinning and jaw fractures [2]. Thus, cyst management is necessary to keep the bone intact and neighboring structures safe. There are two different approaches for treating cysts [2, 3]. The first approach is enucleation of the cyst, which requires the elimination of the cyst completely with its epithelial lining. This approach is used with small cystic cavities that can be enucleated without causing harm to the neighboring vital structures. In the case of cysts, that are in approximation to the borders of mandible, the maxillary sinus, the nasal cavity, or the inferior alveolar canal, the alternative strategy is marsupialization, which requires reducing the internal cystic pressure until the size of the cyst decreases such that it can be easily removed completely without the fear of causing jaw fractures or injuries to neighboring vital structures [2].

The presence of an osseous cavity after cyst enucleation is a clinical challenge that needs to be considered, and attempts to regenerate bone in such an area are pivotal matters of concern [4]. Thus, many studies have evaluated different types of bone grafts, autologous bone, allogenic grafts, xenografts, and synthetic alloplastic materials, in osseous defects to induce bone regeneration and decrease the possibility of infection occurrence [5].

According to the use of autologous grafts, the blood derivatives had gain the potentiality to regenerate tissues in 1990 s, whereas Marx et al. introduced platelet-rich plasma (PRP), the first semi-autologous concentration of platelets. It was used in oral and maxillofacial surgeries in the 1990 s. However, its use did not last long because of the difficult protocol of its preparation, which is performed in 2 stages, and because cross-infection that may occur from the addition of a foreign material such as bovine thrombin [6–8]. Therefore, Choukroun et al. developed a pure autologous concentration of platelets, platelet-rich fibrin (PRF), in 2001. PRF is simply prepared from peripheral venous blood, using plain glass tubes without the addition of blood thinners, which are centrifuged at 3000 revolutions per minute (rpm) for 10 min to produce a fibrin mesh clot with concentrations of platelets, leukocytes, growth factors, and cytokines that promote the healing and regeneration of tissues [7, 9].

Recently, the use of pure autologous concentrations of platelets, PRF, as a graft material after cyst removal has shown promising effects [4]. However, the durability of PRF is limited, as it usually resorbs within 10–14 days; thus, many trials have been performed to change the centrifugation protocol to increase its durability [10–12]. Thus, Mourão et al. introduced a new technique for PRF preparation, in which a venous blood sample is drawn from the patient into a sterile plastic centrifuge tube. The mixture was centrifuged for 8 min at 700 × g, after which the platelet-poor plasma (PPP) layer with 60% albumin was removed and heated at a uniform temperature of 75 $^\circ{\rm C}$ for 10 min to denature the albumin protein, forming an albumin gel. The resulting newly formed protein remains stable for a long time, up to 4 to 6 months. The buffy coat layer of the PRF was then mixed with the denatured albumin gel and cooled to room temperature to form a new autologous albumin gel-platelet-rich fibrin mixture (Alb-PRF) [10, 12–17] which has the ability to heal, represented by the delayed and gradual release of growth factors present in the buffy coat layer of PRF throughout the prolonged dissolution of the albumin gel [10, 14].

As bone regeneration continues after cyst removal, the volume of the cystic cavity decreases. The volume was measured formerly via a panoramic radiograph [18–20], but this method was not accurate, as the panoramic radiograph is a two-dimensional (2D) view [21, 22]. After that, several studies were conducted to measure the three largest diameters of a cystic cavity via cone beam computed tomography (CBCT) scanning and obtain an approximated value for the volume [23]; however, the volume is still not accurately calculated. However, other attempts have been made to measure the 3-dimensional (3D) volumetric change in a cystic cavity by injecting normal saline solution into the cystic defect and calculating the volume of the saline solution used, as mentioned by Yi Zhao et al. [24].

This research aimed to evaluate the effect of an autologous Alb-PRF on bone regeneration after jaw cyst enucleation via 3D volumetric analysis. The null hypothesis of this research was that there would be no significant difference between the group that would be treated by enucleation and Alb-PRF application and that would be treated by enucleation without any additives for bone regeneration.

## Methods

This research used a parallel 1:1 allocation ratio in an open-label randomized controlled clinical trial design and followed the CONSORT reporting guidelines [25]. The participants in this research were selected from cases referred to the Oral and Maxillofacial Surgery Department of the Faculty of Dentistry, Alexandria University from May 2022 to March 2023. The research was registered at ClinicalTrials.gov (Registration ID #NCT05658900) and authorized by the Institutional Review Board of the Research Ethics Committee of the Faculty of Dentistry, Alexandria University, Egypt (International Number IORG0008839; Ethics Committee Number 0414–03/2022). These research activities followed the Declaration of Helsinki for human subjects.

### Sample size calculation

The University of British Columbia's Brant sample size calculator [26] was used to determine the sample size on the basis of Rosner's technique [27]. On the basis of the assumptions of 5% alpha error and 80% research power, the sample size was computed. The mean (SD) percent reduction in lesion volume after 6 months was calculated to be 99.61% (1.06%) after enucleation of cysts without additives [28] and 43.79% (39.84%) for the Alb-PRF group [29]. Using the largest SD of 39.84% to provide sufficient power in the difference between two independent means, the minimum sample size was determined to be 9 participants per group; this was later increased to 10 participants per group.

### Randomization

A computer-generated list of random numbers was used to allocate participants in a parallel 1:1 ratio in blocks of two. The sequence of allocation was concealed in dark-colored envelopes which were distributed sequentially by an independent investigator.

### Aim

The effects of an autologous Alb-PRF on bone regeneration after jaw cyst enucleation were evaluated via 3D volumetric analysis, and the mean bone density was measured postoperative immediately and post 6 months.

### Participants’ eligibility criteria

#### The inclusion criteria

Participants with periapical radicular jaw cysts larger than 1 cm^3^ and smaller than 5 cm^3^.

#### The exclusion criteria [4, 30]

1. Participants with a history of chemotherapy or radiotherapy.
2. Participants with low platelet counts.
3. Participants receiving anticoagulant therapy.
4. Medically compromised participants.

### Interventions

**Fig. 1:**
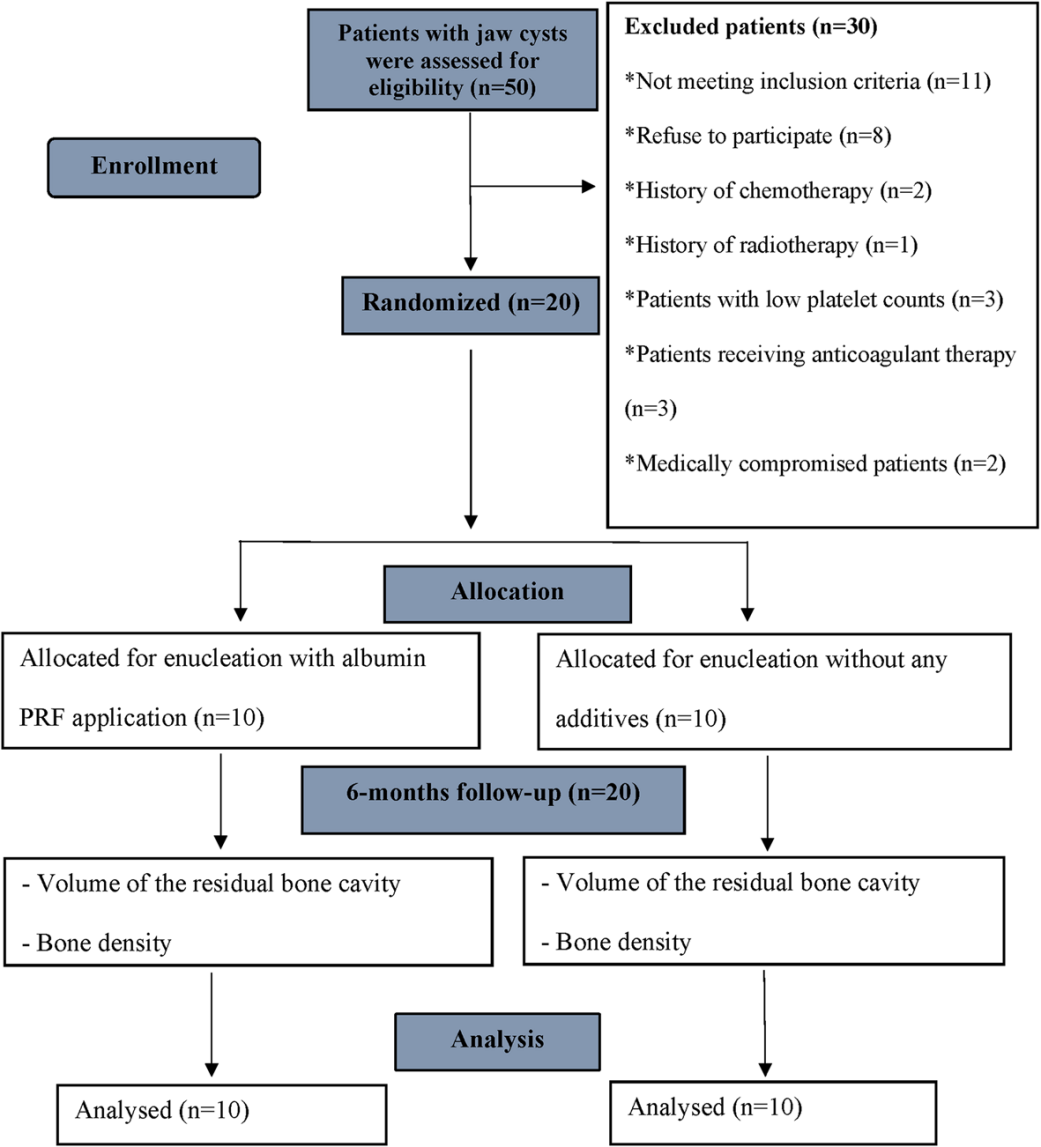
Flow chart showing the participants’ progression in the trial in adherence to the CONSORT guidelines

Two groups, the Alb-PRF group and the control group, each with ten individuals, participated in this research. The Alb-PRF group was managed with enucleation and Alb-PRF application, whereas the control group was managed conventionally with enucleation and without the application of any additives (Fig. 1).

Each participant signed a formal written informed consent form.

### Preoperative assessment and clinical examination

The preoperative assessment included personal, prior dental, and medical records, as well as the patient's main complaint. Upon clinical examination, inspection, palpation, and aspiration were performed. Before surgery, CBCT (T0) was acquired to evaluate the extension of the cystic cavity's lesion, bone density, and volume, and root canal treatment was performed on the teeth that were included in the cystic cavity and presumed to be the cause of the cystic lesion preoperatively.

### Surgical procedures

**Fig. 2:**
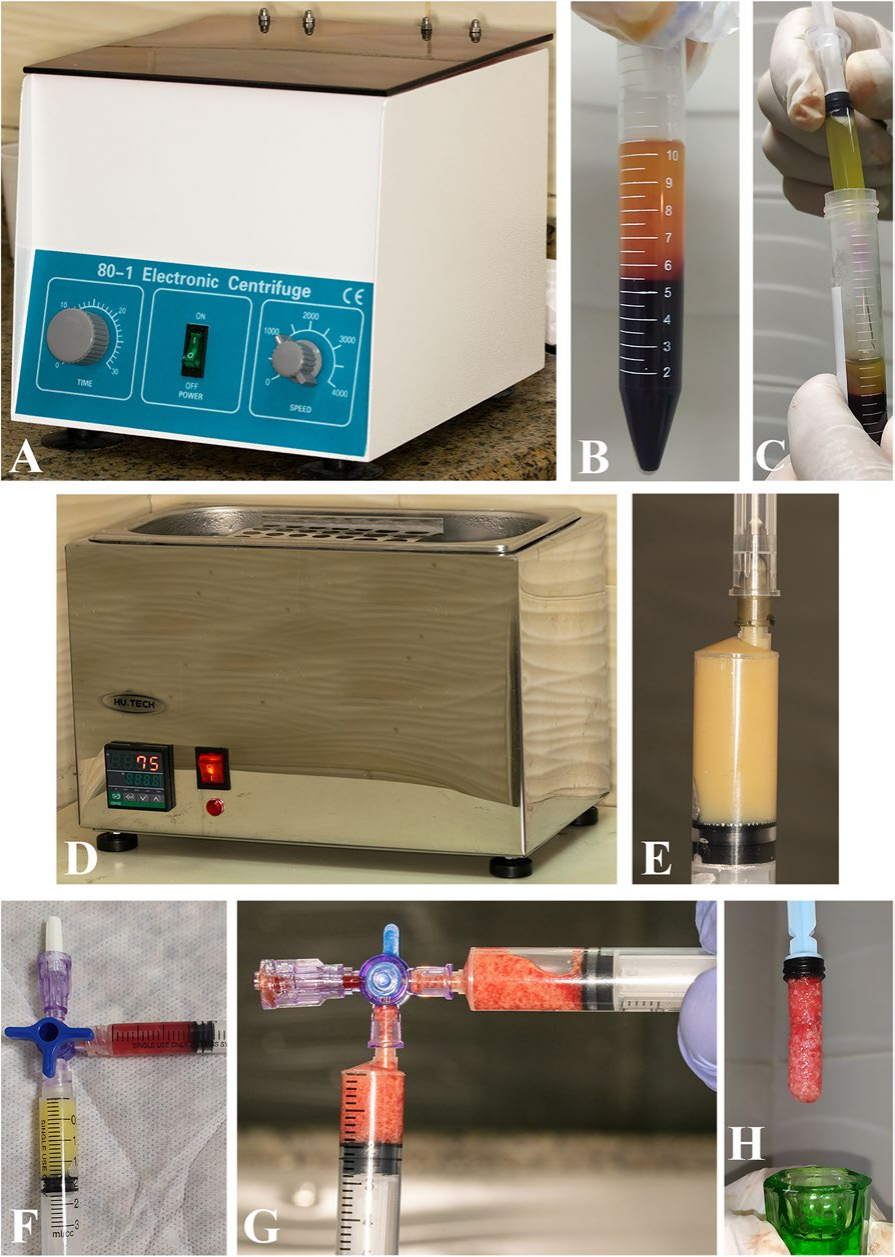
**A** 80–1 centrifuge machine. **B** Venous blood in a sterile plastic centrifuge tube with a blue cap after centrifugation at 700 × g for 8 min. **C** The PPP was removed and heated at a uniform temperature of 75 °C for 10 min using, **D** A Thermostatic water bath incubator. **E** Albumin gel formation after denaturation of the albumin protein. **F **&** G** Albumin gel was mixed with the buffy coat layer of PRF via a 3-way stopcock and 2 plastic syringes with a Luer lock to obtain **H** Albumin PRF

All the necessary laboratory investigations were performed to prepare the patient for surgery. The povidone-iodine surgical scrub solution was swabbed onto the operative site. Depending on the patient's condition, the cystic size, and the scope of the lesion, either local or general anaesthesia was used for surgical treatment. First, a complete-thickness mucoperiosteal flap was created by extending one tooth anteriorly and one tooth posteriorly to the cystic cavity utilizing blades No. 15 and No. 3 Bard-Parker handles. The periosteal elevator was then used to reflect the flap and reveal the bone. The cystic lesion was exposed by removing the surrounding bone, and it was enucleated before being sent for histological analysis. If necessary, apicectomy and retrograde mineral trioxide aggregate (MTA) (Keepers Dent Co., Cairo, Egypt) filling were carried out, in addition to extraction for hopeless teeth. To prepare the Alb-PRF [10, 12, 14, 15], ten milliliters of the patient’s venous blood was withdrawn and collected in a sterile plastic centrifuge tube with a blue cap [31] (Nantong Hailun Bio-Medical Apparatuses Manufacturing Co., Ltd. Haimen City, Jiangsu Province, China), which was subsequently centrifuged at 700 × g for eight minutes via a fixed-angle centrifuge machine (medical laboratory 80–1 electric desktop low-speed centrifuge machine, Chongqing, China). The resultant sample consisted of three layers: red blood cells in the lower, the growth factors, platelets, leukocytes, and cytokines in the buffy coat layer of PRF in the middle, while the PPP was at the top. Using a thermostatic water bath incubator (HU.TECH Co., Alexandria, Egypt), the PPP layer was collected and heated for ten minutes at a constant temperature of 75 °C to denature the albumin protein, forming an albumin gel. After 10 min of cooling to room temperature, the albumin gel was combined with the buffy coat layer of PRF via a 3-way stopcock (ULTRAWAY, Ultra for Medical Products Co., Assiut, Egypt) and two Luer-lock plastic syringes (MASS For Medical Necessities Co., Assiut, Egypt) to create an Alb-PRF (Fig. 2).

**Fig. 3:**
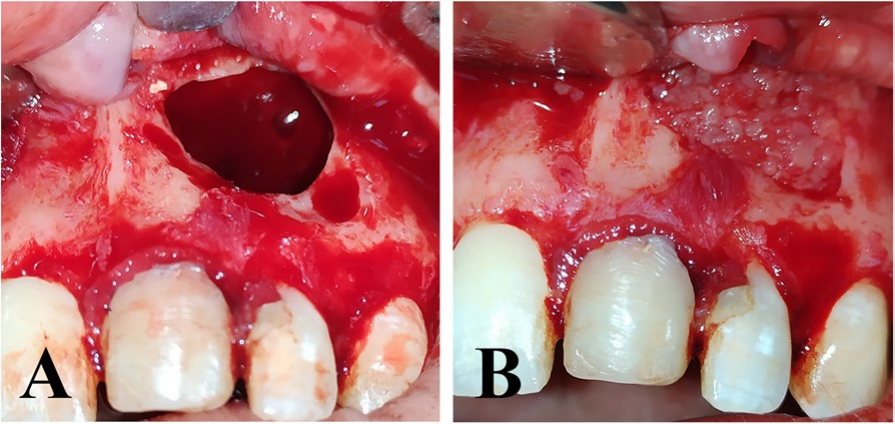
**A** Empty osseous cavity after cyst enucleation. **B** Alb-PRF application inside the osseous cavity. (Alb-PRF group)

The size of the albumin particles was carefully controlled by standardizing the number of times the mixture was passed through the 3-way stopcock, whereas the mixture was passed 10 times to ensure uniformity in particle size distribution. The Alb-PRF group received Alb-PRF (Fig. 3).

**Fig. 4:**
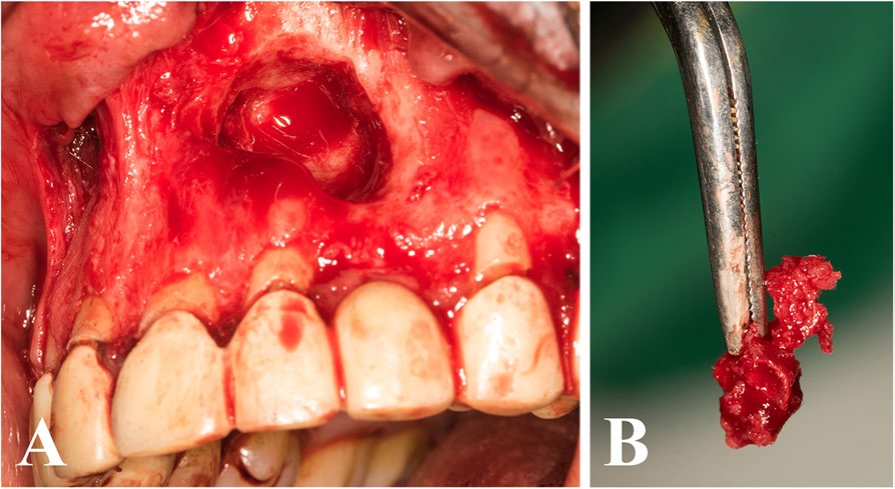
**A** Empty osseous cavity after cyst enucleation. **B** The cyst lining. (Control group)

The control group was treated conventionally without any additives (Fig. 4).

The surgical wound was then closed via 3–0 silk sutures. For a full day, all patients were told to apply ice packs extraorally sporadically for ten minutes every hour. Fluid, soft, high-protein, and high-calorie diets were recommended, whereas hot drinks were avoided on the day of the surgery. All the patients received oral broad-spectrum antibiotics, 875 mg amoxicillin and 125 mg clavulanic acid twice daily (Clavimox 1 g: manufactured by Pharco Co., Egypt), anti-inflammatory and antiedematous medications, chymotrypsin 300 E.A.U and trypsin 300 E.A.U three times daily (Alphintern tablets: manufactured by Amoun Co., Egypt), nonsteroidal anti-inflammatory drugs, diclofenac potassium three times daily (Cataflam 50 mg: manufactured by Novartis, Switzerland), and chlorhexidine hydrochloride twice daily (Hexitol 125 mg: manufactured by The Arab Drug Co., Egypt) for 5 days postoperatively.

### Radiographic follow-up phase

Immediate postoperative CBCT (T1) and six-month postoperative CBCT (T2) were obtained to measure the actual 3D volume of the remaining bone cavity and the average bone density for both groups. The immediate postoperative CBCT (T1) was used as a baseline radiograph of the residual bone cavity immediately after cyst enucleation to show any changes in bone regeneration compared with the post- 6-month CBCT (T2). In our research, CBCT measurements were performed by a single experienced examiner to ensure consistency and minimize variability. To assess measurement reliability, we conducted an intra-examiner reliability analysis, yielding an intra-class correlation coefficient (ICC) of 0.82, indicating good reliability. Additionally, all measurements were performed using standardized imaging protocols and software tools to reduce potential errors.

### To obtain the volume of the residual bone cavity

- ➢ Using Segmentation and fine-tuning threshold technique through On-demand 3D software

**Fig. 5:**
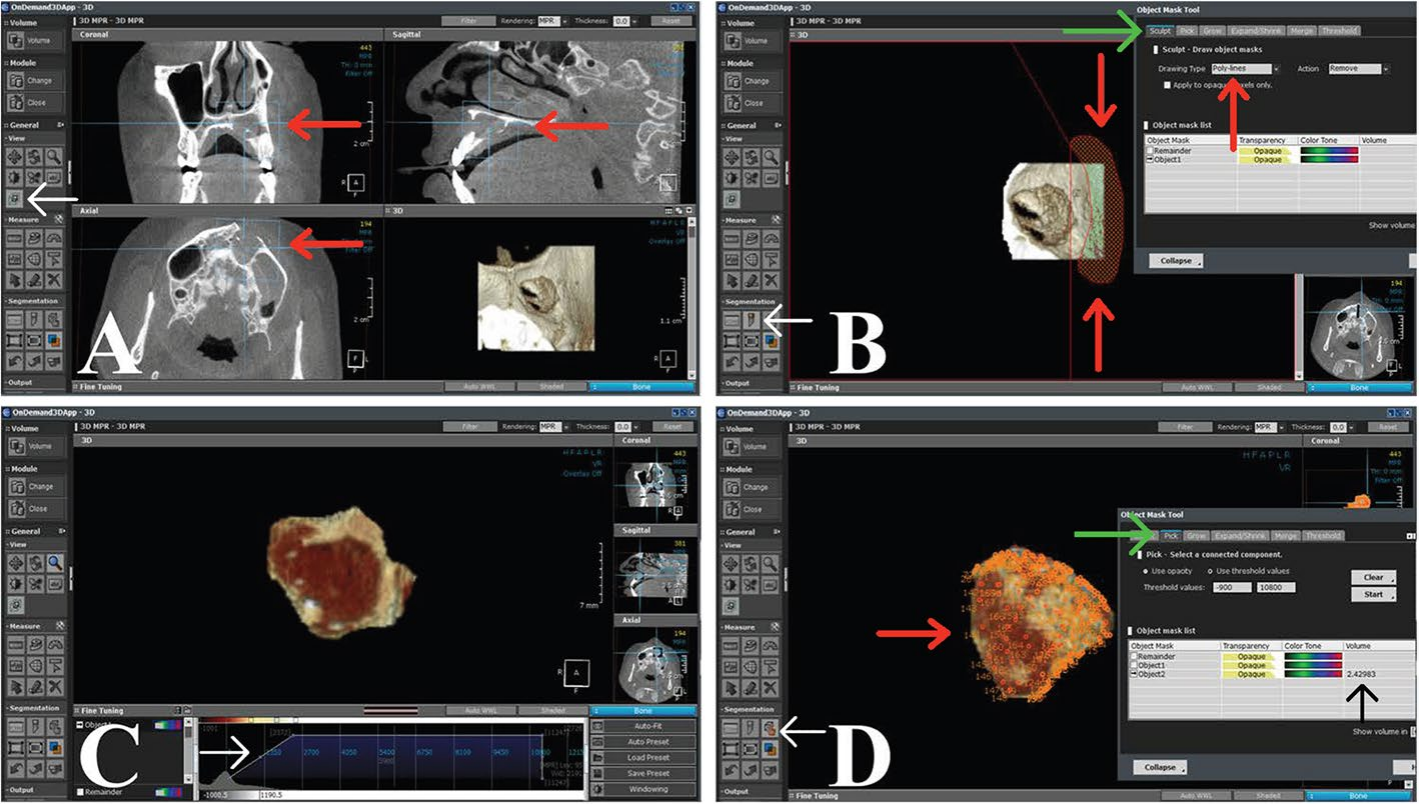
**A** The osseous cavity was cropped via semiautomatic segmentation in coronal, axial, sagittal, and 3D sections. The white arrow refers to the volume of interest (VOI) icon, while the red arrows refer to cropping procedures. **B** The 3D osseous cavity was obtained via manual segmentation. The white arrow refers to the draw mask icon to do manual segmentation, the red arrows refer to the part that will be removed, and the green arrow refers to drawing types. **C** Changing the fine-tuning of the obtained object to be easily detected. The white arrow refers to the fine-tuning threshold scale. **D** Pick up some points on the obtained object to calculate the volume automatically via the On-demand 3D viewer. The white arrow refers to a 3D picker icon, the red arrow refers to picking points on the interested object, and the green arrow refers to the pick icon

The Digital Imaging and Communications in Medicine (DICOM) file for each patient was viewed through On-demand 3D software, and then, with its viewer, each patient's CBCT was illustrated in coronal, axial, sagittal, and 3D sections. First, the osseous cavity was cropped by semiautomatic segmentation in each section; then, manual segmentation was used, the fine-tuning threshold was changed, and the correct 3D osseous cavity was obtained. The volume was subsequently calculated automatically in cubic centimeters (cc) via the On-demand 3D viewer (Fig. 5).

The real 3D volumes of the residual bone cavities at T1 and T2 were compared.

### To obtain the bone density

The DICOM file for each patient was viewed through on-demand 3D software, and then using its viewer, each patient's CBCT was illustrated in coronal, axial, and sagittal sections. The mean density was subsequently measured via the region of interest tool (ROI) at three different positions, standardized in the immediate postoperative CBCT (T1) and after 6 months CBCT (T2) for each patient. The means of the three values for T1 and T2 were subsequently calculated via a grayscale measurement (GV) [32]. The means bone density of T1 and T2 were compared.

### Statistical analysis of data

Data were uploaded into a computer and analysed via the IBM Statistical Package for Social Sciences (SPSS) software version 20.0 (Armonk, NY: IBM Corp). Verifying the distribution's normality was done using the Shapiro–Wilk test. The range (lowest and maximum), mean, and standard deviation, were used to illustrate the quantitative data. The results'significance was assessed at the 5% level. Student t-test and Paired t-test were used to compare normally distributed quantitative variables between two study groups and between two periods, respectively.

## Results

This research involved 20 participants, 9 males, and 11 females, with a mean age of 32.05 years, split into two groups of 10 each: the Alb-PRF group and the control group. The ages of the Alb-PRF group ranged from 22 to 45 years, with a mean age of 33.8 years, whereas the ages of the control group ranged from 21 to 36 years, with a mean age of 30.3 years. Table 1

**Table 1:** Demographic data of the two study groups

	Alb-PRF group (*n* = 10)	Control group (*n* = 10)
Age (years, range)	22—45	21—36
Mean ± **SD**	33.8 ± 7.5	30.3 ± 4.9
Sex, n		
Female	6	5
Male	4	5

*SD* Standard deviation

### The volume of the residual bone cavity

**Fig. 6:**
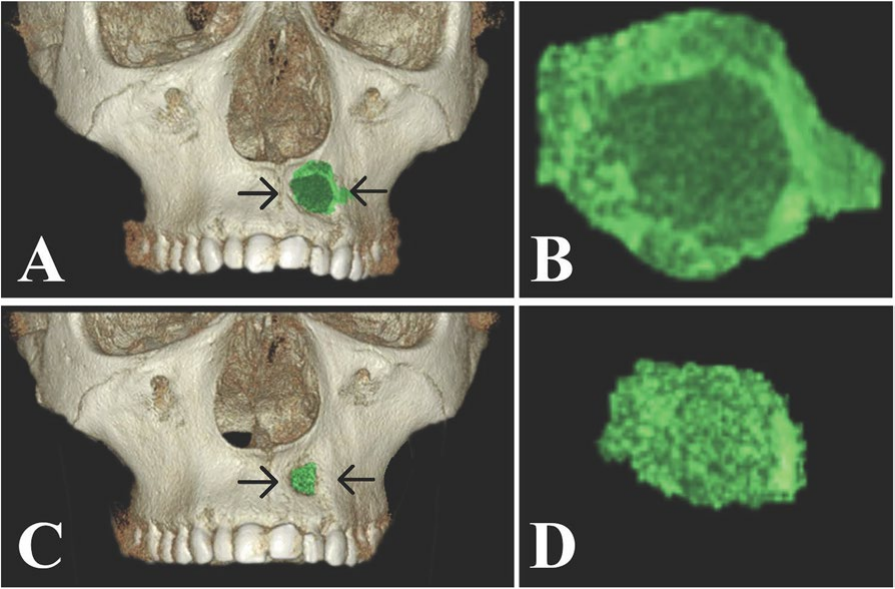
**A** Postoperative immediate 3D reconstruction showing segmentation of the residual bone cavity after cyst enucleation via the on-demand 3D viewer. **B** The segmented postoperative immediate residual bone cavity is used for volume calculation. **C** 3D reconstruction showing the residual bone cavity after 6 months. **D** The segmented residual bone cavity significantly declined in volume after 6 months. The black arrows refer to the segmented residual bone cavity. (Alb-PRF group)

In the Alb-PRF group, the postoperative immediate bone cavity volume ranged from 1.07 cc to 2.74 cc, with a mean of 2.06 $\pm$ 0.71 cc. After 6 months, the bone cavity volume ranged from 0.37 cc to 1.92 cc, with a mean of 1.03 $\pm$ 0.51 cc. A statistically significant decline in residual bone cavity volume was observed in the Alb-PRF group after 6 months (P_1_ < 0.001), MD (95% C.I.) = 1.037 (0.733–1.342). (Table 2; Fig. 6).

**Table 2:** Comparison between the two study groups according to volume

Volume	Alb-PRF group (*n* = 10)	Control group (*n* = 10)	**t**	**p**	MD (95% C.I.)
Postoperative immediate					
Min. – Max	1.07–2.74	1.06–2.49	1.668	0.115	0.447 (-0.123 – 1.016)
Mean ± SD	2.06 ± 0.71	1.62 ± 0.46			
Post 6 months					
Min. – Max	0.37–1.92	0.22–2.18	0.529	0.603	0.136 (-0.403 – 0.675)
Mean ± SD	1.03 ± 0.51	0.89 ± 0.63			
p_1_	< 0.001^*^	< 0.001^*^			
MD (95% C.I.)	1.037 (0.733 – 1.342)	0.727 (0.519 – 0.934)			
% of decline					
Min. – Max	29.67–69.20	12.58–84.92	0.229	0.821	0.311 (-0.032 – 0.653)
Mean ± SD	51.28 ± 12.93	49.30 ± 23.95			

*SD* Standard deviation, *t* Student t-test, *MD* Mean Difference, *CI* Confidence interval, *LL* Lower limit, *UL* Upper Limit

p: *p*-value for comparing between the study groups

p_1_: *p*-value for Paired t-test for comparing between Postoperative immediate and Post 6 months

^*^Statistically significant at *p* ≤ 0.05

**Fig. 7:**
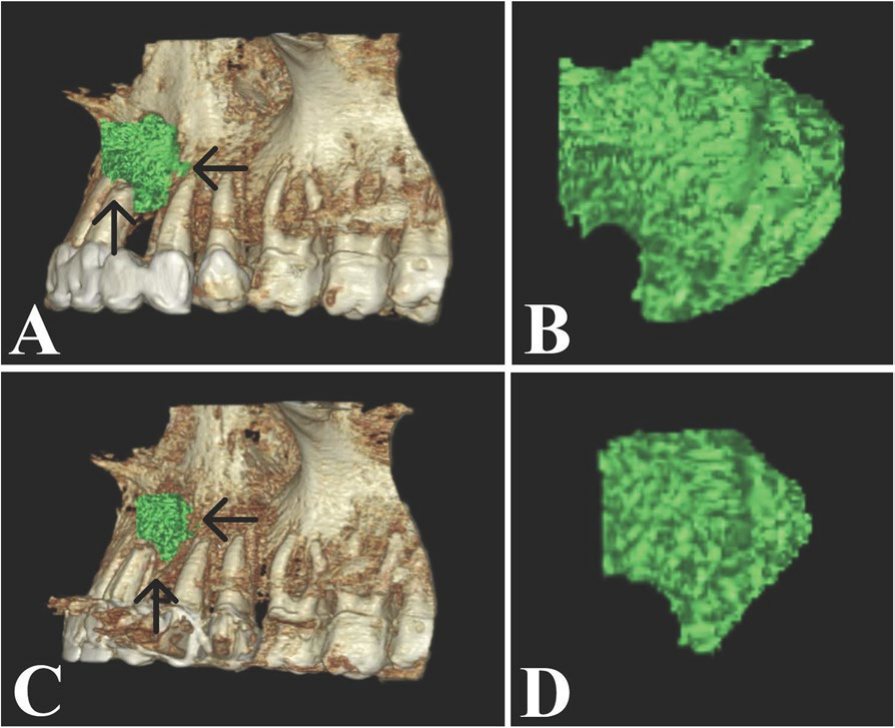
**A** Postoperative immediate 3D reconstruction showing segmentation of the residual bone cavity after cyst enucleation via the on-demand 3D viewer. **B** The segmented postoperative immediate residual bone cavity is used for volume calculation. **C** 3D reconstruction showing the residual bone cavity after 6 months. **D** The segmented residual bone cavity significantly declined in volume after 6 months. The black arrows refer to the segmented residual bone cavity. (Control group)

Within the control group, the postoperative immediate bone cavity volume ranged from 1.06 cc to 2.49 cc, with a mean of 1.62 $\pm$ 0.46 cc. After 6 months, the bone cavity volume ranged from 0.22 cc to 2.18 cc, with a mean of 0.89 $\pm$ 0.63 cc. A statistically significant decline in residual bone cavity volume was observed in the control group after 6 months (P_1_ < 0.001), MD (95% C.I.) = 0.727 (0.519–0.934) (Table 2; Fig. 7).

**Fig. 8:**
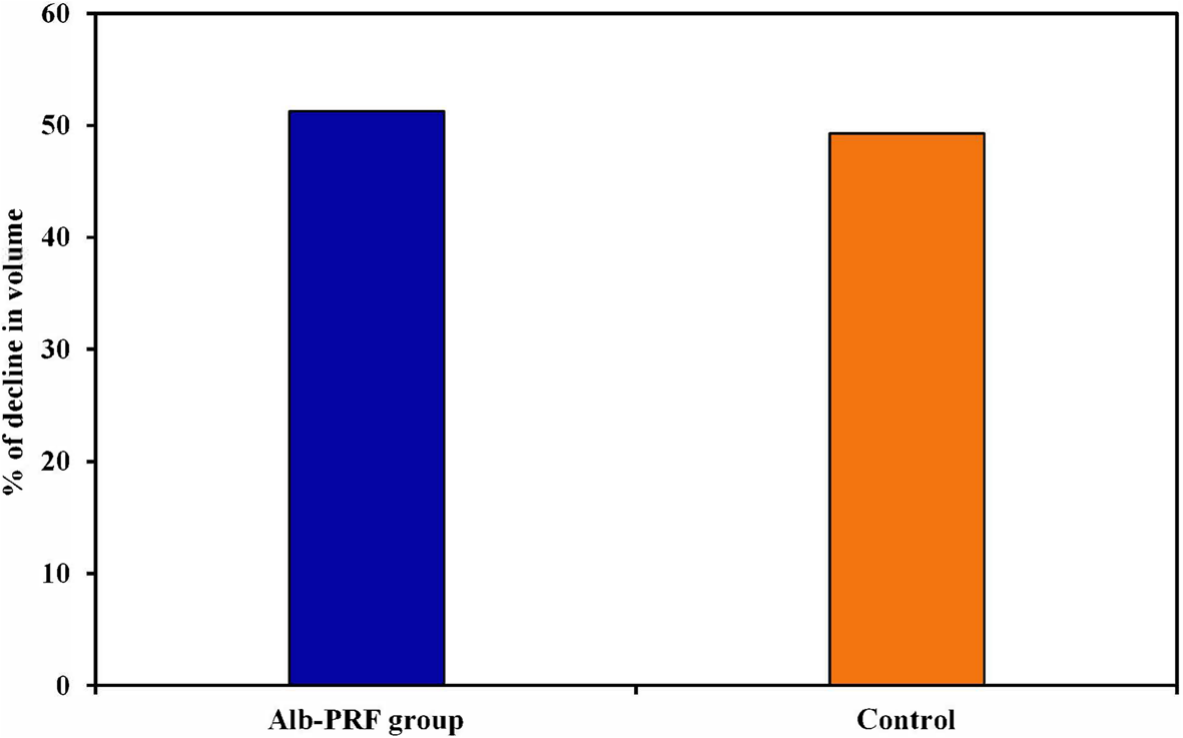
The graph shows the percentage of decline in volume for both groups

Regarding the decline in volume after 6 months in the Alb-PRF group, the percentage of decline ranged from 29.67% to 69.20%, with a mean decline of 51.28 ± 12.93%, whereas within the control group, the percentage of decline in volume ranged from 12.58% to 84.92%, with a mean decline of 49.30 ± 23.95%. Neither of the two groups showed any statistically significant differences after six months, although the Alb-PRF group experienced a greater decline in volume than the control group did (t = 0.229, P = 0.821) MD (95% C.I.) = 0.311 (− 0.032 – 0.653). (Table 2; Fig. 8).

## Bone density

**Fig. 9:**
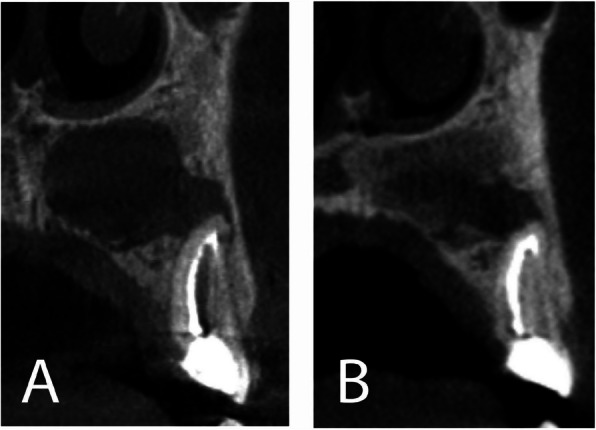
**A** Coronal view showing the residual bone cavity postoperative immediately (T1). **B** Coronal view showing bone formation and a significant increase in bone density after 6 months (T2). (Alb-PRF group)

Regarding the Alb-PRF group, the postoperative immediate bone density ranged from − 145.9 GV to 114.6 GV, with a mean of − 54.81 90.11 GV. After 6 months, the bone density ranged from 7.26 GV to 376.3 GV, with a mean of 220.9 134.1 GV. Statistical analysis revealed an increase in bone density in the Alb-PRF group after 6 months (P_1_ < 0.001). (Table 3; Fig. 9)

**Table 3:** Comparison between the two study groups according to density

Density	Alb-PRF group (*n* = 10)	Control group (*n* = 10)	**t**	**p**	MD (95% C.I.)
Postoperative immediate					
Min. – Max	− 145.9 – 114.6	− 107.3 – 159.0	0.676	0.508	28.257 (-59.626 –116.140)
Mean ± SD	− 54.81 ± 90.11	− 26.34 ± 96.98			
Post 6 months					
Min. – Max	7.26–376.3	101.63–233.0	1.279	0.225	59.016 (-41.346 – 159.378)
Mean ± SD	220.9 ± 134.1	161.9 ± 57.47			
p_1_	< 0.001^*^	< 0.001^*^			
Increase					
Min. – Max.	79.29 – 487.5	57.66 – 336.0	1.497	0.152	87.273 (-35.185 – 209.731)
Mean ± SD.	275.5 ± 158.0	188.2 ± 94.99			
% of Increase					
Min. – Max	110.1–719.1	39.52–417.0	0.636	0.533	44.858 (-103.278–192.993)
Mean ± SD	305.9 ± 185.5	261.0 ± 123.7			

*SD* Standard deviation, *t* Student t-test, *MD* Mean Difference, *CI* Confidence interval, *LL* Lower limit, *UL* Upper Limit

p: *p*-value for comparing between the study groups

p_1_: *p*-value for Paired t-test for comparing between Postoperative immediate and Post 6 months

^*^: Statistically significant at *p* ≤ 0.05

**Fig. 10:**
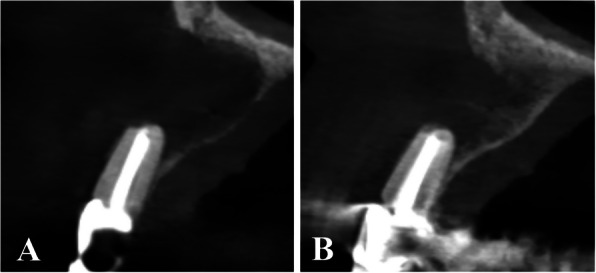
**A** Sagittal view showing the residual bone cavity postoperative immediately (T1). **B** Sagittal view showing bone formation and a significant increase in bone density after 6 months (T2). (Control group)

Within the control group, the postoperative immediate bone density ranged from − 107.3 GV to 159.0 GV, with a mean of − 26.34 $\pm$ 96.98 GV. After 6 months, the bone density ranged from 101.63 GV to 233.0 GV with a mean of 161.9 $\pm$ 57.47 GV. After six months, the bone density of the control group increased (P_1_ < 0.001). (Table 3; Fig. 10).

**Fig. 11:**
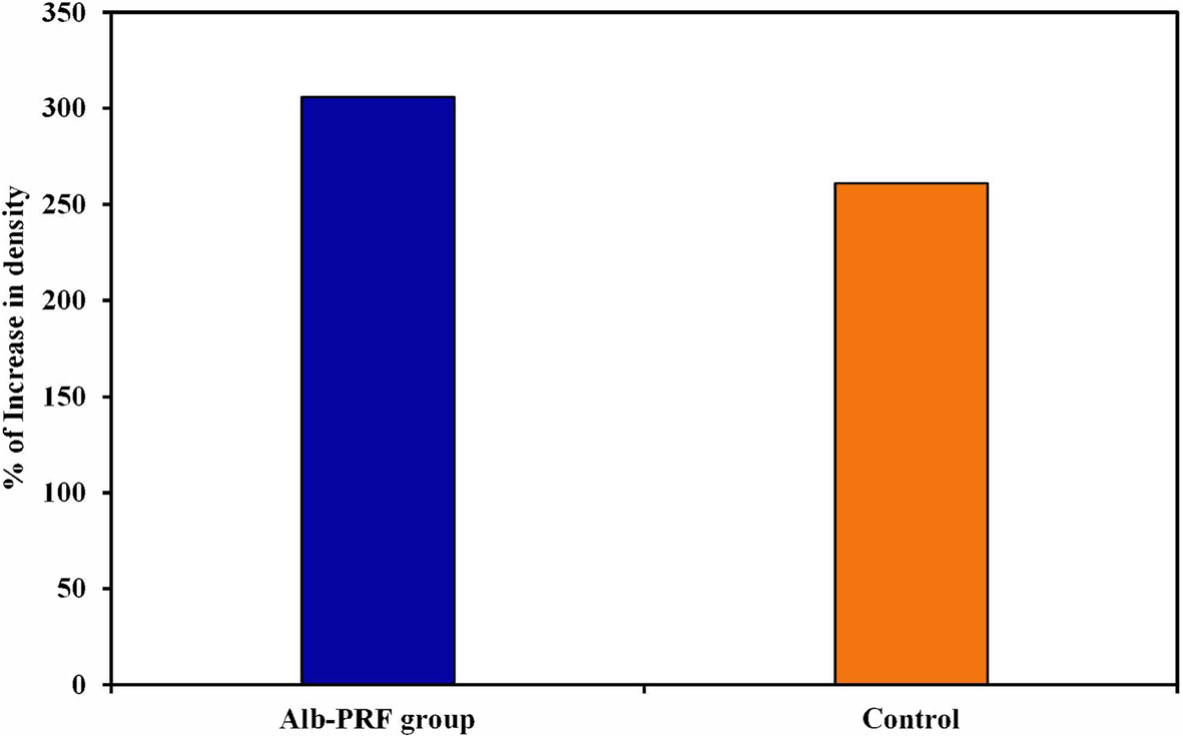
The graph shows the percentage of increase in density for both groups

Regarding the increase in bone density after 6 months within the Alb-PRF group, the percentage of increase ranged from 110.1% to 719.1%, with a mean increase of 305.9 ± 185.5%, whereas within the control group, the percentage of increase ranged from 39.52% to 417.0%, with a mean increase of 261.0 ± 123.7%. While the Alb-PRF group experienced a greater increase in bone density than did the control group after six months, the two groups did not significantly differ (t = 0.636, *P* = 0.533), MD (95% C.I.) = 44.858 (− 103.278–192.993). (Table 3; Fig. 11).

## Discussion

The presence of an osseous cavity after cyst enucleation is a clinical challenge that needs to be considered. Several studies suggest filling the osseous cavity with a filler material to promote healing, whereas others consider spontaneous healing of the osseous cavity without adding a promoting filler material as the standard approach [2].

This research aimed to evaluate the effect of an autologous Alb-PRF on bone regeneration after jaw cyst enucleation via 3D volumetric analysis.

CBCT and the On-demand 3D viewer were used in this research to demonstrate the osseous cavity in three dimensions and to measure its volume and the density of the regenerative bone, in accordance with Schloss et al., who recommended the use of CBCT rather than 2D periapical films to illustrate the volume of periapical lesions accurately [33]. In contrast, previous studies had used the panoramic radiograph [18–20], a 2D view, which was not accurate enough to measure the 3D volume [21, 22]. According to Ahlowalia et al., CBCT is an accurate tool for measuring the volume of osseous bovine cavities in vivo [34]. Similar to our research, Abdel-Ghany H et al. used CBCT to demonstrate the volume of the cystic cavity in three dimensions via DICOM files and the segmentation tools of Planmeca Romexis software [35], while Al-Qurmoti et al. used CBCT and Image J software to measure the jaw cystic cavity volume following enucleation [36]. In corresponding with our research regarding the volume measurement, You et al. used CBCT and Mimics software to segment and evaluate the volume of the radicular lesions in upper anterior teeth following endodontic surgery [37]. In this research, the preoperative CBCT (T0) was conducted to evaluate the volume, the extension of cystic lesion, and its relation with neighboring vital structures preoperatively, but the immediate postoperative CBCT (T1) was conducted and used as a baseline radiograph of the residual bone cavity immediately after cyst enucleation where during surgery the cyst was enucleated and any undermined necrotic bone was removed leaving intact healthy bone walls, so the accurate volume of residual bone cavity and the bone density could be measured and compared with the post- 6-month CBCT (T2). CBCT has advantages over computed tomography (CT). Using CBCT lessens the patient's needless exposure and the amount of dispersed radiation that could deteriorate image quality. CBCT is cost-effective and segmental, so no need for high exposure radiation. The reliability of measuring bone density using CBCT is questionable. In the past, CT was a reasonable radiograph for measuring bone density and counting Hounsfield units (HU); however, high radiation dosage, expense, accessibility, and extended scanning duration have led to the use of CBCT instead [38]. The ability to consider the benefits and risks of using which radiograph is necessary, so whether to choose a reasonable bone density measurement with high radiation dosage and high cost, or a less reliable bone density measurement with low radiation dosage and less cost. Nowadays, the orientation towards using CBCT in measuring bone density has been common [39].

This research revealed that the volume of the residual bone cavity had declined, whereas the density of regenerative bone at the osseous cavity had increased significantly after 6 months in the Alb-PRF group compared with postoperative immediate results (P_1_ < 0.001). The volume declined from 2.06 ± 0.71 cc to 1.03 ± 0.51 cc with a mean decline of 51.28 ± 12.93% after 6 months. While the density increased from − 54.81 ± 90.11 GV to 220.9 ± 134.1 GV, with a mean increase of 305.9 ± 185.5% after 6 months. This was attributed to the ability of Alb-PRF to heal, represented by the delayed and gradual release of growth factors present in the buffy coat layer of PRF throughout the prolonged dissolution of the albumin gel, which can remain stable for a long time, up to 4 to 6 months [10, 12–14]. The extended biodegradability of Alb-PRF was described by E. Gheno et al., who reported a longer duration of stability of Alb-PRF than horizontal platelet-rich fibrin (H-PRF) and leukocyte platelet-rich fibrin (L-PRF) [12]. This resorption period is based on existing literature and preliminary observations [10]; thus, it still needs more studies to make sure that Alb-PRF remains stable up to 4 to 6 months. Similarly, M. Fujioka-Kobayashi et al. conducted an in vitro study via an enzyme-linked immunosorbent assay (ELISA) test. These authors characterized seven growth factors from Alb-PRF, including transforming growth factor beta 1 (TGF-$\beta 1$), epidermal growth factor (EGF), insulin growth factor 1 (IGF- 1), vascular endothelial growth factor (VEGF), and 3 forms of platelet-derived growth factor (PDGF) [10]. These growth factors play important roles in the regeneration of tissues. They are released from platelets at the fibrin mesh of Alb-PRF and then bind to their receptors on target cell membranes, stimulating intracellular messages to express genes involved in the healing process [40]. The potential of Alb-PRF in the healing and regeneration of tissues was illustrated clinically by Leonida et al., who reported promising effects of Alb-PRF when they used activated plasma albumin gel (APAG) in elevating the maxillary antrum through the dental socket [15], and by Javid et al., who reported similar results regarding the potential of Alb-PRF in the healing and regeneration of tissues when they used Alb-PRF in dental sockets after extraction of lower third molars [16].

On the other hand, in our control group, the statistical analysis revealed that the volume of the residual bone cavity had declined, whereas the density of regenerative bone at the osseous cavity had increased significantly after 6 months without the use of any filler material compared with the immediate postoperative results (P_1_ < 0.001). This research revealed that the volume had declined from 1.62 ± 0.46 cc to 0.89 ± 0.63 cc with a mean decline of 49.30 ± 23.95% after 6 months. While the density had increased from − 26.34 ± 96.98 GV to 161.9 ± 57.47 GV, with a mean increase of 261.0 ± 123.7% after 6 months. These results corresponded with earlier studies performed by Wagdargi et al. and Rubio, Mombru` [41, 42]. The presence of negative values for bone density measurements postoperatively immediately in both groups illustrates the poor quality of bone and the presence of some air postoperatively immediately. These negative values corresponded with the new classification for bone mineral density mentioned by Xiao et al. [39]; however, after 6 months, the bone density measurements were positive, which illustrates the regeneration of bone and increased density and minerals. Wagdargi et al. reported that the mean reduction in the size of the osseous cavity after cyst removal was 60%, whereas the mean increase in bone density was 90.2% after 6 months without the use of any filler material [41]. Similarly, Chiapasco et al. conducted a study to estimate the self-regeneration of bone and reported that the mean reduction in the volume of the cystic cavity was 12.3%, whereas the mean increase in the density of regenerative bone was 37.0% after 6 months [5]. According to Chacko et al., concerning the spontaneous healing of the osseous cavity after the eradication of jaw cysts, the mean decreases in the size of the osseous cavity were 25.85% and 100% after 6 months and 2 years, respectively [18]. Al-Qurmoti et al. revealed that the decrease in the volume of the residual bone cavity was rapid in the first 6 months and then slowed in the next period after that [36].

Several authors consider the removal of cysts and closure of mucoperiosteal flaps over the osseous cavity while keeping the periosteum and endosteum and the maximum number of bone walls intact surrounding osseous defects the gold benchmark manoeuvres for spontaneous regeneration of bone without the use of any filler material. The bone regeneration procedure is a complex mechanism that is maintained through several factors, such as the blood clot and growth factors, with intact periosteum, endosteum, and a maximum number of residual bone walls [5, 42, 43]. According to Rubio, Mombru`, the blood clot is the best bioactive filler material to be used inside an osseous cavity. Its stabilization is mandatory to help in the healing process, so intact residual bone walls are necessary to preserve it inside the osseous cavity [42].

In this research, when the Alb-PRF group and the control group were compared, the decline in the volume of the residual bone cavity and the increase in the density of regenerative bone were greater in the Alb-PRF group than in the control group; nevertheless, no statistically significant difference in bone regeneration was found between the two groups. Similarly, some studies had been conducted in the literature and revealed that the autologous concentration of platelets had no significant effect on healing. Sharma et al. reported that there was no statistically meaningful distinction between PRF growth factors and conventional wound recovery following tooth extraction in terms of bone maturation and bone density [44]. Kumar YR et al. concluded results similar to those of our study when they compared conventional PRF healing to conventional blood clot healing in a control group after tooth extraction [45]. Considering our findings, You et al. revealed no statistically significant variation among PRF, concentrated growth factor, and control conventional blood clots after 6 months in terms of the osseous defect reduction rate [37]. These findings suggest that the growth factors, whether their source is Alb-PRF, conventional PRF, PRP, or conventional blood clots, are the key regulators of the bone regeneration process.

However, Dar et al. reported a significant effect of autologous PRF on the density and healing of bone within 6 months after cyst enucleation, despite the absence of a control group [4]. In contrast, Salman Shams et al. reported that PRP, a blood derivative, has significant potential for bone regeneration after cyst enucleation, where the mean defect bone fill was 95.95% at the 24 th week [46]. Moreover, A. A. Alzahrani et al. reported promising effects of PRF on socket healing after extraction, where the mean radiographic bone fill was noticeably greater in the PRF group (88.81 ± 1.53%) than in the control group (80.35 ± 2.61%) after 2 months [47].

Alb-PRF is one of the PRF derivatives and is still not widely tested clinically, so there are not enough trials in the literature that have clinically investigated Alb-PRF. According to M. Fujioka-Kobayashi et al., who investigated Alb-PRF in vitro at the cellular level, Alb-PRF has the potential to regenerate tissues, whereas Alb-PRF induces cell mitotic division and enhances the viability of cells experimentally, while E. Gheno et al. managed to reveal an extended in vivo stability period for Alb-PRF and a prolonged release of growth factors, which could enhance the clinical practice of Alb-PRF. It is an autologous material that can be used safely without fear of any antibody reactions. So, we hope to use Alb-PRF in the future as a barrier membrane in guided tissue regeneration (GTR) or as a filler regenerative material in any bone defects [10, 12]. Variations in albumin particle size could potentially influence the study's outcomes, particularly concerning bone formation. To further investigate this, histological studies may be required to assess the impact of albumin particle size on bone tissue formation at a microscopic level, which is one of our research limitations.

The relatively small sample size of 20 participants may limit the ability to detect significant differences between the Alb-PRF and control groups, and we have addressed this as a research limitation. However, this sample size was determined based on findings from previous studies and was sufficiently powered to detect changes over time within each group. Since not enough prior studies have investigated Alb-PRF, this study serves as an initial exploration, providing a foundation for future research. We recommend further studies with larger sample sizes to validate our findings and better assess differences between groups.

The limited performance of Alb-PRF in our research may be due to some limitations of this research, whereas the absence of histological evaluation, the limited sample size, and the insufficient duration of follow-up were not enough to estimate the regenerative potential of Alb-PRF after cyst enucleation or give an advantage to it. Thus, additional clinical studies with a large number of participants and an extended period of follow-up associated with histological evaluation are recommended. Another limitation of this research is that it was retrospectively registered, which could introduce bias that may affect our research results and transparency. This limitation could have implications for the design and planning of our study, so we suggest and recommend the prospective registration for future studies, which will improve transparency and reduce the potential for bias.

## Conclusion

According to our research, Alb-PRF and conventional blood clots were not significantly different in terms of residual bone cavity volume reduction or the density of the newly formed bone, although Alb-PRF is one of the PRF derivatives. Alb-PRF is safe and cost-effective to use as a filler material without fear of causing infection because it is a pure autologous material. The non-significant results may be due to the limited sample size and the insufficient duration of follow-up.

## Data Availability

The data used and analysed in this study are available from the corresponding author upon reasonable request.

## Abbreviations

PRF: Platelet-rich fibrin
Alb-PRF: Albumin gel-platelet-rich fibrin mixture
CBCT: Cone beam computed tomography
PRP: Platelet-rich plasma
MTA: Mineral trioxide aggregate
RPM: Revolutions per minute
PPP: Platelet poor plasma
DICOM: Digital Imaging and Communications in Medicine
ROI: Region of interest
SPSS: Statistical Package for Social Sciences
CC: Cubic centimeter
GV: Gray value
ELIZA: Enzyme-linked immunosorbent assay
TGF-${\varvec{\beta}}1$: Transforming growth factor beta 1
EGF: Epidermal growth factor
IGF- 1: Insulin growth factor 1
VEGF: Vascular endothelial growth factor
PDGF: Platelet-derived growth factor
CT: Computed tomography
HU: Hounsfield unit
H-PRF: Horizontal platelet-rich fibrin
L-PRF: Leukocyte platelet-rich fibrin
APAG: Activated plasma albumin gel
GTR: Guided tissue regeneration
